# High‐resolution HLA class II sequencing of Swedish multiple sclerosis patients

**DOI:** 10.1111/iji.12594

**Published:** 2022-08-12

**Authors:** Omar Akel, Lue Ping Zhao, Daniel E Geraghty, Alexander Lind

**Affiliations:** ^1^ Department of Clinical Sciences Malmö Clinical Research Centre Lund University, Skåne University Hospital SUS Malmö Sweden; ^2^ Clinical Research Division Fred Hutchinson Cancer Research Center Seattle WA United States

**Keywords:** autoimmunity, high‐resolution sequencing, human leukocyte antigen, multiple sclerosis

## Abstract

Multiple sclerosis (MS) is a chronic neurological disease believed to be caused by autoimmune pathogenesis. The aetiology is likely explained by a complex interplay between inherited and environmental factors. Genetic investigations into MS have been conducted for over 50 years, yielding >100 associations to date. Globally, the strongest linkage is with the human leukocyte antigen (HLA) HLA‐DRB5*01:01:01‐DRB1*15:01:01‐DQA1*01:02:01‐DQB1*06:02:01 haplotype.

Here, high‐resolution sequencing of HLA was used to determine the alleles of *DRB3*, *DRB4*, *DRB5*, *DRB1*, *DQA1*, *DQB1*, *DPA1* and *DPB1* as well as their extended haplotypes and genotypes in 100 Swedish MS patients. Results were compared to 636 population controls.

The heterogeneity in HLA associations with MS was demonstrated; among 100 patients, 69 extended *HLA‐DR‐DQ* genotypes were found. Three extended *HLA‐DR‐DQ* genotypes were found to be correlated to MS; HLA‐DRB5*01:01:01‐DRB1*15:01:01‐DQA1*01:02:01‐DQB1*06:02:01 haplotype together with

(A) HLA‐DRB4*01:01:01//DRB4*01:01:01:01‐DRB1*07:01:01‐DQA1*02:01//02:01:01‐DQB1*02:02:01,

(B) HLA‐DRBX*null‐DRB1*08:01:01‐DQA1*04:01:01‐DQB1*04:02:01, and

(C) HLA‐DRB3*01:01:02‐DRB1*03:01:01‐DQA1*05:01:01‐DQB1*02:01:01.

At the allelic level, HLA‐DRB3*01:01:02 was considered protective against MS. However, when combined with HLA‐DRB3*01:01:02‐DRB1*03:01:01‐DQA1*05:01:01‐DQB1*02:01:01, this extended haplotype was considered a predisposing risk factor. This highlights the limitations as included with investigations of single alleles relative to those of extended haplotypes/genotypes.

In conclusion, with 69 genotypes presented among 100 patients, high‐resolution sequencing was conducted to underscore the wide polymorphisms present among MS patients. Additional studies in larger cohorts will be of importance to define MS among the patient group not associated with HLA‐DRB5*01:01:01‐DRB1*15:01:01‐DQA1*01:02:01‐DQB1*06:02:01.

## 1 INTRODUCTION

Multiple sclerosis (MS) is the most common inflammatory disease affecting the central nervous system. Epidemiological findings suggest a gene–environment interaction, and the pathogenesis of MS is widely assumed to be of autoimmune origin (Filippi et al., 2018). Overall, the strongest genetic factor associated with MS is the human leukocyte antigen (HLA)‐DRB1*15:01 allele (Allen et al., 1994; Mack et al., 2019; Marrosu et al., 2001; Sawcer et al., 2005; Schmidt et al., 2007). Among individuals with European origin, HLA‐DRB1*15:01 is inherited in almost complete linkage disequilibrium (LD) with HLA‐DQB1*06:02. Studies in African‐Americans, among whom the HLA‐DQB1*06:02 allele is not found on HLA‐DRB1*15:01 haplotypes, has attributed the risk of MS to HLA‐DRB1*15:01. Additional *HLA*‐DRB1‐DQB1 alleles and haplotypes associated with MS include

- HLA‐DRB1*03:01‐DQA1*05:01‐DQB1*02:01 (Mack et al., 2019; Marrosu et al., 2001; Marrosu et al., 1998; Moutsianas et al., 2015; Sanna et al., 2010; The International Multiple Sclerosis Genetics Consortium & The Wellcome Trust Case Control Consortium 2, 2011),
- HLA‐DRB1*04:05‐DQA1*05:01‐DQB1*0301 (Marrosu et al., 2001; Marrosu et al., 1998),
- HLA‐DRB1*04:05‐DQB1*03:02 (Marrosu et al., 2001; Moutsianas et al., 2015),
- HLA‐DRB1*13:03‐DQB1*03:01 (Marrosu et al., 2001; Moutsianas et al., 2015; The International Multiple Sclerosis Genetics Consortium & The Wellcome Trust Case Control Consortium 2, 2011) and
- *H* LA‐DRB1*08:01 (Barcellos et al., 2006; Moutsianas et al., 2015).

A recent family‐study used high‐resolution sequencing to report a novel predisposing association to MS for HLA‐DPB1*104:01, independent of HLA‐DRB1*15:01 haplotype. High‐resolution sequencing also conferred negative MS association to HLA‐DRB1*01:01:01, HLA‐DQB1*03:01 and HLA‐DQB1*03:03 (Osoegawa et al., 2021) and HLA‐DRB1*14:04:01 (Vinoy et al., 2021).

Secondary HLA‐DRB loci, including *HLA‐DRB3*, *DRB4* and *DRB5*, have been less defined for MS. HLA‐DRB5*01:01 is found in linkage disequilibrium with HLA‐DRB1*15:01 and therefore found in increased frequency among MS patients. Both HLA‐DRB5*01:01 and HLA‐DRB1*15:01 present peptides in heterodimer pocket‐formation with non‐polymorphic HLA‐DRα. HLA‐DRB5*01:01 has been indicated to modulate the presumed disease promoting HLA‐DRB1*15:01 antigen‐presentation since rare HLA‐DRB5*null haplotypes among HLA‐DRB1*15:03 African‐American MS patients correlated with an increased risk of developing severe secondary progressive MS (Caillier et al., 2008).

Sweden has one of the highest prevalence of MS worldwide (Ahlgren et al., 2014; Ahlgren et al., 2012), partly explained by the high frequency of the HLA‐DRB1*15:01‐DQB1*06:02 haplotype in the population.

The aim of the studies was to define HLA genetics among MS patients not associated with HLA‐DRB5*01:01:01‐DRB1*15:01:01‐DQA1*01:02:01‐DQB1*06:02:01. Here, we used high‐resolution sequencing to determine the associations between MS and *HLA*‐*DRB3*, *DRB4*, *DRB5*, *DRB1*, *DQA1*, *DQB1*, *DPA1* and *DPB1*. This kind of typing offers substantial advantages over methods of imputation and, therefore, more accurately captures the polymorphic complexity of the HLA region.

## 2 METHODS AND MATERIALS

### 2.1 Study population

The MS Malmö cohort (*n* = 100) included blood samples collected from the Neurology Department of the Skåne University Hospital in Malmö. The MS diagnosis of the patients was validated by treating physician (**Table** **1**) (McDonald et al., 2001; Thompson et al., 2018). Blood samples were collected in cell preparation tubes (CPTs). Controls (*n* = 636) included in the HLA Next Generation Sequencing (NGS) analysis were obtained as previously described (Lind et al., 2019). These controls were obtained as two different study groups: (A) Swedish general population (GP) controls (*n* = 448) and (B) LifeGene controls (*n* = 188; https://www.lifegene.se/For‐scientists/About‐LifeGene) (Lind et al., 2019; Zhao et al., 2016). Sample collection and HLA typing was performed at different time‐points; the two different control groups were verified to be harmonize (no statistical differences for any HLA alleles, haplotypes or genotypes) before put together as one larger group of general population controls. General population controls refer to a large enough and randomly selected group of individuals, which represent the HLA genotypes present in the Swedish population.

**TABLE 1:** Clinical and demographical data

	**MS patients**
*n*	100
Gender	Female *n* = 63, Male *n* = 36
Age at sampling, median years (range)	40 (24‐66)
Age at diagnosis, median years (range)	30 (16‐60)
Subtype of MS	Relapsing‐remitting MS *n* = 92, Secondary progressive MS *n* = 8

The study was approved by the regional ethics board in Lund, Sweden (dnr 2015/257 with amendment 2017/670).

### 2.2 HLA high‐resolution typing

DNA was extracted using Qiagen Blood Maxi Kit according to manufacturer's protocol and as previously described (Lind et al., 2019). In brief, blood clots were lysed through addition of protease (Qiagen protease) and lysis buffer (AL buffer, Qiagen). Next, incubation of sample‐mixtures was at 70°C for 10 min. Addition of 99.5% ethanol was followed by sample‐mixture addition to binding‐columns (QIAamp maxi columns). Centrifugation was at 4000 rpm for 3 min. The columns were washed with buffers (AW1 followed by AW2, Qiagen) and the DNA was extracted by addition of elution buffer (Buffer AE, Qiagen).

HLA NGS was performed by Scisco Genetics (Seattle, USA). Samples (50 ng/μl, 20 μl) were sent blindly to the investigators. HLA NGS makes use of PCR‐based HLA amplification and sequencing with Illumina MiSeq technology, as described in previous studies (Nelson et al., 2015; Smith et al., 2014; Zhao et al., 2016). The alleles of *HLA‐DRB1*, ‐*DRB345*, ‐*DQA1*, ‐*DQB1*, ‐*DPA1* and ‐*DPB1* were used to compute the extended haplotypes for each individual on the basis of family structures detailed in previous narcolepsy family studies and the most common haplotype frequency among individuals with European origin (Johansson et al., 2008; Lampis et al., 2000; Papassavas et al., 2000; Reil et al., 1997; Skibola et al., 2012). The population controls in the study were sequenced at two different time points [first occasion: Swedish general population (GP) controls (*n* = 448), and second occasion: LifeGene controls (*n* = 188)]; in the meantime the resolution of typing was improved; therefore, that some alleles are written as follows in the manuscript DRB4*01:01:01//DRB4*01:01:01:01.

### 2.3 Statistics

Calculations for HLA NGS analysis were performed using R‐studios and the function ‘haplo.cc’ (http://cran.r‐project.org/web/packages/haplo.stats/index.html), as previously described (Lind et al., 2019). The significance threshold for all hypothesis testing was unadjusted and set to .05. Correction for multiple testing was applied using the Benjamini–Hochberg Procedure (Benjamini & Hochberg, 1995).

## 3 RESULTS

### 3.1 HLA‐DRB3, DRB4 and DRB5‐DRB1‐DQA1‐DQB1 genotypes

A total of 95 *HLA‐DRB3*, *DRB4* and *DRB5‐DRB1‐DQA1‐DQB1* genotypes were present among MS patients or GP (Supplementary Table S1). Among 100 MS patients, 69 different genotypes were found. Relative to GP controls, a higher frequency of three genotypes were found in MS patients. Notably, all these three genotypes included a single HLA‐DRB5*01:01:01‐DQB1*06:02:01 haplotype:

1. HLA‐DRB5*01:01:01‐DRB1*15:01:01‐DQA1*01:02:01‐DQB1*06:02:01;
DRBX*null‐DRB1*08:01:01‐DQA1*04:01:01‐DQB1*04:02:01,
2. HLA‐DRB5*01:01:01‐DRB1*15:01:01‐DQA1*01:02:01‐DQB1*06:02:01;
HLA‐DRB3*01:01:02‐DRB1*03:01:01‐DQA1*05:01:01‐DQB1*02:01:01,
3. HLA‐DRB5*01:01:01‐DRB1*15:01:01‐DQA1*01:02:01‐DQB1*06:02:01; DRB4*01:01:01//DRB4*01:01:01:01‐DRB1*07:01:01‐DQA1*02:01//02:01:01‐DQB1*02:02:01 (**Table** **2**).

**TABLE 2:** Estimated HLA‐DR ‐DQ genotypic and haplotypic frequencies in MS patients and GP controls, estimated odds ratios (OR), estimated H‐score (HS) measuring associations and associated *p* values

	**Control *n* (%)**	**MS *n* (%)**	**OR**	**HS**	** *p* Value**
DRB5*01:01:01‐DRB1*15:01:01‐DQA1*01:02:01‐DQB1*06:02:01; DRB3*01:01:02‐DRB1*03:01:01‐DQA1*05:01:01‐DQB1*02:01:01	19 (2.7)	7 (7.0)	1.93	2.26	2.35E‐02
DRB5*01:01:01‐DRB1*15:01:01‐DQA1*01:02:01‐DQB1*06:02:01; DRB4*01:01:01//DRB4*01:01:01:01‐DRB1*07:01:01‐DQA1*02:01//02:01:01‐DQB1*02:02:01	5 (0.8)	6 (6.0)	3.29	3.99	6.48E‐05
DRB5*01:01:01‐DRB1*15:01:01‐DQA1*01:02:01‐DQB1*06:02:01; DRBX*null‐DRB1*08:01:01‐DQA1*04:01:01‐DQB1*04:02:01	7 (0.9)	5 (5.0)	2.74	3.11	1.88E‐03
DRB3*02:02:01‐DRB1*03:01:01‐DQA1*05:01:01‐DQB1*02:01:01	19 (1.7)	9 (4.5)	4.43	2.62	8.84E‐03
DRB4*01:03:01‐DRB1*04:02:01‐DQA1*03:01:01‐DQB1*03:02:01	6 (0.5)	6 (3.0)	10.58	3.71	2.06E‐04
DRB4*01:03:01‐DRB1*04:03:01‐DQA1*03:01:01‐DQB1*03:02:01	8 (0.5)	5 (2.5)	5.53	2.64	8.26E‐03
DRB5*01:01:01‐DRB1*15:01:01‐DQA1*01:02:01‐DQB1*06:02:01	194 (15.3)	55 (27.5)	2.75	4.28	1.90E‐05
DRB5*02:02//DRB5*02:02:01‐DRB1*16:01:01‐DQA1*01:02:02‐DQB1*05:02:01	14 (1.1)	8 (4.0)	3.65	3.03	2.47E‐03
DRBX*null‐DRB1*01:02:01‐DQA1*01:01:02‐DQB1*05:01:01	4 (0.3)	5 (2.5)	12.46	3.70	2.18E‐04
DRB4*01:03:01‐DRB1*07:01:01‐DQA1*02:01//*02:01:01‐DQB1*03:03:02	31 (2.5)	0		−2.25	2.41E‐02

### 3.2 HLA‐DRB3, DRB4 and DRB5‐DRB1‐DQA1‐DQB1 haplotypes

In total, in the study, 122 different *HLA‐DRB3*, *DRB4* and *DRB5‐DRB1‐DQA1‐DQB1* haplotypes were found. The complete results and associations for all the different haplotypes found in the study are reported in Supplementary Table S2.

A reference haplotype with a similar frequency between the study groups was selected in HLA DRB3*03:01:01‐DRB1*13:02:01‐DQA1*01:02:01‐DQB1*06:04:01 (4.0% and 3.5%).

Based on the Haplo‐Score, **6** haplotypes with positive (A) and **1** haplotypes with negative (B) association for MS patients in comparison to population controls were identified (presented here in numerical order):

- Identified haplotypes with higher frequency among MS patients compared to GP were (1) HLA‐DRB3*02:02:01‐DRB1*03:01:01‐DQA1*05:01:01‐DQB1*02:01:01, (2) HLA‐DRB4*01:03:01‐DRB1*04:02:01‐DQA1*03:01:01‐DQB1*03:02:01, (3) HLA‐DRB4*01:03:01‐DRB1*04:03:01‐DQA1*03:01:01‐DQB1*03:02:01, (4) HLA‐DRB5*01:01:01‐DRB1*15:01:01‐DQA1*01:02:01‐DQB1*06:02:01, (5) HLA‐DRB5*02:02//DRB5*02:02:01‐DRB1*16:01:01‐DQA1*01:02:02‐DQB1*05:02:01 and (6) HLA‐DRBX*null‐DRB1*01:02:01‐DQA1*01:01:02‐DQB1*05:01:01.
- Identified haplotypes with lower frequency among MS patients compared to GP were (1) HLA‐DRB4*01:03:01‐DRB1*07:01:01‐DQA1*02:01//*02:01:01‐DQB1*03:03:02 (Table 2).

### 3.3 Alleles

In total, in the study, 18 different *HLA‐DRB3*, *DRB4*, and *DRB5* alleles, 47 different *HLA‐DRB1* alleles, 23 different *HLA‐DQA1* alleles, 24 different *HLA‐DQB1* alleles, 14 *HLA*‐*DPA1* and 30 *HLA‐DPB1* alleles were found. The complete results and associations for all the different alleles found in the study are reported in Supplementary Table S3. Selected alleles with a comparable frequency in patients and controls were used as reference. Those alleles included HLA‐DRB3*03:01:01 (4.0%, 4.4%), *HLA‐* DRB1*03:01:01 (11.5%, 12.0%), *HLA‐*DQA1*03:01:01 (12.5%, 12.7%), HLA DQB1*02:01:01 (11.5%, 11.3%), *HLA*‐DPA1*01:03:01 (84.3%, 84.0%) and HLA‐DPB1*02:01:02 (14.0%, 14.5%). Based on the Haplo‐Score, 9 alleles with positive and 7 alleles with negative association for MS patients in comparison to population controls were identified (Table 3).

**TABLE 3:** Estimated allelic frequencies of HLA‐DRB3, DRB4, DRB5, ‐DRB1, ‐DQA1, ‐DQB1, ‐DPA1 and ‐DPB1, estimated odds ratios (OR) of individual alleles, estimated H‐score (HS) measuring associations and associated *p* values

	**Control *n* (%)**	**MS *n* (%)**	**OR**	**HS**	***p* Value**
*HLA* ‐*DRB3*, *DRB4*, *DRB5*					
DRB3*01:01:02	204 (16.0)	18 (9.0)	0.64	−2.58	9.83E‐03
DRB4*01:03:01	307 (24.1)	32 (16.0)	0.76	−2.52	1.17E‐02
DRB5*01:01:01	205 (16.1)	63 (31.5)	2.30	5.25	1.52E‐07
DRB5*02:02// DRB5*02:02:01	16 (1.3)	8 (4.0)	3.38	2.75	5.88E‐03
HLA‐*DRB1*					
DRB1*04:02:01	7 (0.6)	6 (3.0)	4.45	3.46	5.45E‐04
DRB1*13:01:01	92 (7.2)	5 (2.5)	0.32	−2.57	1.02E‐02
DRB1*15:01:01	202 (15.9)	61 (30.5)	1.99	5.03	4.94E‐07
DRB1*16:01:01	16 (1.3)	8 (4.0)	2.81	2.75	5.88E‐03
HLA‐*DQA1*					
DQA1*01:01:01	160 (12.6)	9 (4.5)	0.43	−3.20	1.38E‐03
DQA1*01:01:02	4 (0.3)	5 (2.5)	9.21	3.70	2.18E‐04
DQA1*01:02:01	265 (20.8)	70 (35.0)	1.85	4.46	8.15E‐06
DQA1*01:02:02	18 (1.4)	9 (4.5)	3.15	2.94	3.28E‐03
DQA1*01:03:01	101 (7.9)	8 (4.0)	0.53	−2.02	4.35E‐02
*HLA‐DQB1*					
DQB1*03:03:02	49 (3.9)	1 (0.5)	0.14	−2.38	1.75E‐02
DQB1*05:02:01	16 (1.3)	12 (6.0)	4.36	4.30	1.71E‐05
DQB1*06:02:01	201 (15.8)	56 (28.0)	1.64	4.21	2.52E‐05
DQB1*06:03:01	98 (7.7)	7 (3.5)	0.37	−2.19	2.87E‐02

## 4 DISCUSSION

For simplified reading of the discussion section, significant haplotypes are presented as the following abbreviations:

- **HLA‐DR15‐DQ6** is HLA‐DRB5*01:01:01‐DRB1*15:01:01‐DQA1*01:02:01‐DQB1*06:02:01;
- HLA‐DRB3*01‐DR3‐DQ2 is HLA‐DRB3*01:01:02‐DRB1*03:01:01‐DQA1*05:01:01‐DQB1*02:01:01;
- HLA‐DRB3*02‐DR3‐DQ2 is HLA‐DRB3*02:02:01‐DRB1*03:01:01‐DQA1*05:01:01‐DQB1*02:01:01;
- **HLA‐DR7‐DQ2** is HLA‐DRB4*01:01:01//DRB4*01:01:01:01‐DRB1*07:01:01‐DQA1*02:01//02:01:01‐DQB1*02:02:01 and
- **HLA‐DR8‐DQ4** is HLA‐DRBX*null‐DRB1*08:01:01‐DQA1*04:01:01‐DQB1*04:02:01.

From the present study, we first report that previously found HLA‐associations to MS, most prominently to *DR15‐DQ6*, can be confirmed in 100 MS patients from Southern Sweden. The three genotypes with increased frequency in MS included the *HLA‐DR15‐DQ6* haplotype together with previously reported HLA‐DRB1*08:01 (*HLA‐DR8‐DQ4*), HLA‐DRB3*01‐*DR3‐DQ2*, while the association with *HLA‐DR7‐DQ2* appear novel.

Here, we could not confirm recent allelic MS associations using high‐resolution sequencing to HLA‐DPB1*104:01 (predisposing), HLA‐DQB1*03:01 and HLA‐DQB1*03:03 (protective), and HLA‐DRB1*14:04:01 (protective) (Osoegawa et al., 2021; Vinoy et al., 2021). These alleles were not found to be commonly present in the Swedish population, with exception of HLA‐DQB1*03:01. In our study, we found HLA‐DQB1*03:01 on the extended HLA DRB3*02:02:01‐DQA1*05:05:01 haplotype, with various combinations of *DRB1*‐alleles (*11:01:01, 11:04:01, 12:01:01, 13:05:01*). Due to high degree of polymorphism presented, we suggest that mechanistic studies would be necessary to fully determine the role of HLA‐DQB1*03:01 to risk and progression of MS.

While HLA‐DRB3*01:01:02 was considered protective against MS at the allelic level, the extended genotype, abbreviated *HLA‐DR15‐DQ6*‐DRB3*01‐DR3‐DQ2, was considered a predisposing risk factor. This highlights the limitations of studies of single alleles relative to studies of extended haplotypes and genotypes. We speculate that the shifting association for HLA‐DRB3*01:01:02 from protective to predisposing, could be related to mechanisms of epistasis. Previously, HLA‐DQA1*01:02 was demonstrated to increase risk of MS if found in trans position to the haplotype of *HLA‐DR15‐DQ6* (Lincoln et al., 2009).

Lastly, we report heterogeneity in HLA associations to MS given that, among 100 patients, 69 different extended *HLA‐DR‐DQ* genotypes were identified. Approximately half of all genotypes (36 of 69) and two‐thirds of all patients (68 of 100) encompassed haplotypes of either ***HLA‐DR15‐DQ6***, HLA‐DRB3*01‐DR3‐DQ2 or HLA‐DRB3*02‐DR3‐DQ2.

A substantial number of studies over the last 50 years have associated HLA genetics to MS (Hollenbach & Oksenberg, 2015). The extended *HLA‐DR15‐DQ6* haplotype is consistently reported, both here and elsewhere, as the major genetic contributor to MS. Some suggestions of the presence of this extended haplotype at the age at onset (Hensiek et al., 2002; Masterman et al., 2000; The International Multiple Sclerosis Genetics Consortium & The Wellcome Trust Case Control Consortium 2, 2011), but not during the clinical course of MS, have been suggested (Barcellos et al., 2006). Although a dose effect may explain the data (Barcellos et al., 2006; Barcellos et al., 2003), our study failed to corroborate this finding, as the prevalence of *HLA‐DR15‐DQ6* homozygosity in Swedish population controls was found to be fairly high [2.5% (16/636 controls) as compared to 6% (6/100 MS patients]. There are conflicting reports as to whether the predisposition to MS stems from the *HLA‐DR* or *DQ* region. For example, strong linkage disequilibrium within the HLA‐II region among European populations has made it difficult to establish separate influences of DRB1*1501 and DQB1*0602 in the extended *HLA‐DR15‐DQ6* haplotype. Studies with inclusion of African American populations with diverse patterns of linkage disequilibrium found a selective association to DRB1*1501, independent of DQB1*0602 (McElroy et al., 2010; Oksenberg et al., 2004).

The group of non‐*DR15‐DQ6* associated MS patients (44 out of 100 patients) is of interest, complementary triggers and pathways of autoimmunity could be present. It cannot be excluded that environmental factors are different between MS patients negative for ***HLA‐DR15‐DQ6*** but positive for ***HLA‐DR7‐DQ2***. Studies in larger and diversified cohorts would be needed to define MS in this group of patients.

Historically, the HLA‐DRB1*03:01 allele has been associated with MS. An increased risk was reported when the HLA‐DRB1*03:01 allele is homozygous (Moutsianas et al., 2015). In contrast our study found a similar frequency at the allelic level (patients 11.5%–12.0% controls), and there were no MS homozygous patients present [MS patients; 0/100 (0%) and controls; 10/636 (1.6%)]. Combining *HLA‐DRB1* and *HLA‐DRB345* alleles into haplotypes revealed a risk associated with HLA‐DRB3*02‐DR3‐DQ2, while HLA‐DRB3*01‐DR3‐DQ2 was associated with MS at a genotypic level. This exemplifies that risk associations and protective associations do not necessarily agree at the allelic, haplotypic and genotypic level.

The total of six MS‐associated extended *HLA‐* *DR‐DQ* haplotypes found in the studies means that other haplotypes than *DR15‐DQ6* may contribute to MS. However, combined into genotypes, three MS‐associated variants were found in the *HLA‐DR15‐DQ6* haplotype combined with *HLA‐DR8‐DQ4*, HLA‐DRB3*01‐DR3‐DQ2, *HLA‐DR7‐DQ2*. A plausible explanation for this outcome is the greater depth afforded by high‐resolution sequencing, causing a more accurate reflection of the wide polymorphism found in the HLA region. Another plausible explanation is a less dominant effect of a few specific HLA genes. The notion of smaller influences by many genes has been supported by low large‐family linkage studies (Sawcer et al., 2014). The high‐resolution HLA typing makes it possible to further dissect the possible contribution of other alleles, perhaps haplotypes, to the risk of MS.

A weakness of our study was the fact that the *HLA‐DPA1* and *HLA‐DPB1* alleles could not be assigned to extended haplotypes and genotypes. This was largely attributed to the very high frequency of HLA‐DPA1*01:03:01 (>80%) among both patients and controls, which precluded computation of the other rare allele variants among the families that were used as templates (Lind et al., 2019). Another limitation was the missing information of age at first symptom and family history of MS, these parameters would have been of importance especially among non‐*HLA‐DR15‐DQ6* patients.

It appears overly simplistic to characterize MS as having a single aetiology and pathogenesis. The heterogeneous clinical presentation, variable clinical course, inconsistency in genetic markers, unpredictable therapeutic response and diverse histopathological findings may be indicative of divergence in the demyelination pathways (Lucchinetti et al., 2000; McFarland & Martin, 2007). The high‐resolution HLA as presented here would represent an important approach to better delineate MS aetiology and pathogenesis in the future.

In summary, we have confirmed previous HLA associations to MS, while also offering plausible explanations to help guide future research. Using high‐resolution sequencing, we have highlighted the widespread polymorphisms as 69 distinct genotypes present among 100 Swedish MS patients and further studies in a larger number of patients will be needed to further delineate MS not associated with *HLA‐DR15‐DQ6.2*.

## CONFLICT OF INTEREST

There are no conflicts of interest.

## Supporting information

Supplement Material

Supplement Material
