# Supplementary material for: High‐resolution HLA class II sequencing of Swedish multiple sclerosis patients
Source: Int J Immunogenet. 2022 Aug 12;49(5):333–9. doi: 10.1111/iji.12594 (PMC9545082; doi:10.1111/iji.12594)
Supplement: Supplementary file 1 — Supplement Material [file IJI-49-333-s001.docx]

| Supplementary Table 1. Estimated HLA-*DR -DQ* genotypic frequencies in MS patients and GP controls, estimated odds ratios (OR), estimated H-score (HS) measuring associations, and associated P-values. Genotype variants marked in light grey (higher) and dark grey (lower) frequency differed between MS patients and controls. | | | | | | |
| --- | --- | --- | --- | --- | --- | --- |
| Gen | HLA-DR and DQ genotypes | Control n (%) | MS n (%) | OR | HS | P-value |
| Base | DRB5*01:01:01-*15:01:01-*01:02:01-*06:02:01;DRB4*01:03:01-*04:01:01-*03:01:01-*03:02:01 | 19 (2.8) | 2 (2.0) | 1.00 | 0.47 | 6.35E-01 |
| 1 | DRB5*01:01:01-*15:01:01-*01:02:01-*06:02:01;DRB3*01:01:02-*03:01:01-*05:01:01-*02:01:01 | 19 (2.7) | 7 (7.0) | 1.93 | 2.26 | 2.35E-02 |
| 2 | DRB5*01:01:01-*15:01:01-*01:02:01-*06:02:01;DRB3*02:02:01-*03:01:01-*05:01:01-*02:01:01 | 2 (0.2) | 1 (1.0) |  |  |  |
| 3 | DRB5*01:01:01-*15:01:01-*01:02:01-*06:02:01;DRB3*02:02:01-*11:01:01-*05:05:01-*03:01:01 | 9 (1.3) | 1 (1.0) | 1.06 | 0.22 | 8.27E-01 |
| 4 | DRB5*01:01:01-*15:01:01-*01:02:01-*06:02:01;DRB3*02:02:01-*11:04:01-*05:05:01-*03:01:01 | 3 (0.3) | 2 (2.0) | 3.00 | 2.13 | 3.31E-02 |
| 5 | DRB5*01:01:01-*15:01:01-*01:02:01-*06:02:01;DRB3*02:02:01-*12:01:01-*05:05:01-*03:01:01 | 7 (0.9) | 1 (1.0) |  |  |  |
| 6 | DRB5*01:01:01-*15:01:01-*01:02:01-*06:02:01;DRB3*02:02:01-*13:01:01-*01:03:01-*06:03:01 | 7 (1.1) | 0 |  | –1.05 | 2.92E-01 |
| 7 | DRB5*01:01:01-*15:01:01-*01:02:01-*06:02:01;DRB3*02:02:01-*13:01:01-*01:03:01-*06:03:01 | 0 | 2 (2.0) |  | 3.57 | 3.55E-04 |
| 8 | DRB5*01:01:01-*15:01:01-*01:02:01-*06:02:01;DRB3*03:01:01-*13:02:01-*01:02:01-*06:04:01 | 10 (1.6) | 2 (2.0) | 1.34 | 0.31 | 7.54E-01 |
| 9 | DRB5*01:01:01-*15:01:01-*01:02:01-*06:02:01;DRB4*01:01:01//DRB4*01:01:01:01-*07:01:01-*02:01//02:01:01-*02:02:01 | 5 (0.8) | 6 (6.0) | 3.29 | 3.99 | 6.48E-05 |
| 10 | DRB5*01:01:01-*15:01:01-*01:02:01-*06:02:01;DRB4*01:03:01-*04:01:01-*03:02//03:03:01-*03:01:01 | 6 (0.8) | 3 (3.0) | 2.32 | 1.98 | 4.72E-02 |
| 11 | DRB5*01:01:01-*15:01:01-*01:02:01-*06:02:01;DRB4*01:03:01-*04:02:01-*03:01:01-*03:02:01 | 1 (0.2) | 1 (1.0) |  |  |  |
| 12 | DRB5*01:01:01-*15:01:01-*01:02:01-*06:02:01;DRB4*01:03:01-*04:03:01-*03:01:01-*03:02:01 | 1 (0.2) | 3 (3.0) | 5.20 | 3.59 | 3.25E-04 |
| 13 | DRB5*01:01:01-*15:01:01-*01:02:01-*06:02:01;DRB4*01:03:01-*04:04:01-*03:01:01-*03:02:01 | 6 (0.9) | 2 (2.0) | 1.73 | 0.95 | 3.44E-01 |
| 14 | DRB5*01:01:01-*15:01:01-*01:02:01-*06:02:01;DRB4*01:03:02-*04:04:01-*03:01:01-*03:02:01 | 1 (0.2) | 1 (1.0) |  |  |  |
| 15 | DRB5*01:01:01-*15:01:01-*01:02:01-*06:02:01;DRB5*01:01:01-*15:01:01-*01:02:01-*06:02:01 | 16 (2.5) | 6 (6.0) | 1.84 | 1.90 | 5.72E-02 |
| 16 | DRB5*01:01:01-*15:01:01-*01:02:01-*06:02:01;DRB5*01:01:01-*15:01:01-*01:02:01-*06:03:01 | 0 | 1 (1.0) |  |  |  |
| 17 | DRB5*01:01:01-*15:01:01-*01:02:01-*06:02:01;DRBX*null-*01:01:01-*01:01:01-*05:01:01 | 18 (2.8) | 0 |  | –1.70 | 8.85E-02 |
| 18 | DRB5*01:01:01-*15:01:01-*01:02:01-*06:02:01;DRBX*null-*08:01:01-*04:01:01-*04:02:01 | 7 (0.9) | 5 (5.0) | 2.74 | 3.11 | 1.88E-03 |
| 19 | DRB5*01:01:01-*15:01:01-*01:02:01-*06:02:01;DRBX*null-*08:01:01-*04:02-*04:02:01 | 0 | 1 (1.0) |  |  |  |
| 20 | DRB5*01:01:01-*15:01:01-*01:02:01-*06:02:01;DRBX*null-*08:03:02-*01:03:01-*06:01:01 | 0 | 1 (1.0) |  |  |  |
| 21 | DRB5*01:01:01-*15:01:01-*01:02:01-*06:02:01;DRBX*null-*10:01:01-*01:05:01-*05:01:01 | 0 | 1 (1.0) |  |  |  |
| 22 | DRB5*01:01:01-*15:03:01-*01:02:01-*06:02:01;DRB3*01:01:02-*03:01:01-*05:01:01-*02:01:01 | 0 | 1 (1.0) |  |  |  |
| 23 | DRB5*01:01:01-*15:01:01-*01:02:02-*05:02:01;DRB3*01:01:02-*13:01:01-*01:03:01-*06:03:01 | 0 | 1 (1.0) |  |  |  |
| 24 | DRB5*01:01:01-*15:01:01-*01:02:01-*05:02:01;DRB4*01:03:01-*04:02:01-*03:01:01-*03:02:01 | 0 | 1 (1.0) |  |  |  |
| 25 | DRB5*01:01:01-*15:01:01-*01:02:01-*05:02:01;DRB5*02:02//DRB5*02:02:01-*16:01:01-*01:02:02-*05:02:01 | 0 | 1 (1.0) |  |  |  |
| 26 | DRB5*01:01:01-*15:01:01-*01:02:01-*06:187;DRB5*02:02//DRB5*02:02:01-*16:01:01-*01:02:02-*05:02:01 | 0 | 1 (1.0) |  |  |  |
| 27 | DRB5*01:01:01-*15:01:01-*01:02:01-*06:03:01;DRB3*02:02:01-*11:04:01-*05:05:01-*03:01:01 | 0 | 1 (1.0) |  |  |  |
| 28 | DRB5*01:01:01-*15:06:01-*01:02:01-*05:02:01;DRBX*null-*01:02:01-*01:01:02-*05:01:01 | 0 | 1 (1.0) |  |  |  |
| 29 | DRB3*01:01:02-*03:01:01-*05:01:01-*02:01:01;DRB3*01:01:02-*13:01:01-*01:03:01-*06:03:01 | 6 (0.9) | 1 (1.0) |  |  |  |
| 30 | DRB3*01:01:02-*03:01:01-*05:01:01-*02:01:01;DRB3*02:02:01-*11:01:01-*05:05:01-*03:01:01 | 8 (1.3) | 1 (1.0) | 1.06 | 0.22 | 8.27E-01 |
| 31 | DRB3*01:01:02-*03:01:01-*05:01:01-*02:01:01;DRB3*03:01:01-*13:02:01-*01:02:01-*06:09//*06:09:01 | 0 | 1 (1.0) |  |  |  |
| 32 | DRB3*01:01:02-*03:01:01-*05:01:01-*02:01:01;DRB4*01:03:01-*04:01:01-*03:01:01-*03:02:01 | 8 (1.3) | 1 (1.0) | 1.06 | 0.22 | 8.27E-01 |
| 33 | DRB3*01:01:02-*03:01:01-*05:01:01-*02:01:01;DRB4*01:03:01-*04:04:01-*03:01:01-*03:02:01 | 9 (1.4) | 0 |  | –1.20 | 2.31E-01 |
| 34 | DRB3*01:01:02-*03:01:01-*05:01:01-*02:01:01;DRBX*null-*01:01:01-*01:01:01-*05:01:01 | 17 (2.5) | 1 (1.0) | 0.75 | 0.94 | 3.48E-01 |
| 35 | DRB3*01:01:02-*03:01:01-*05:01:01-*02:01:01;DRBX*null-*08:01:01-*04:01:01-*04:02:01 | 4 (0.6) | 1 (1.0) |  |  |  |
| 36 | DRB3*01:01:02-*13:01:01-*01:03:01-*06:03:01;DRB5*01:02-*15:02:01-*01:03:01-*06:01:01 | 0 | 1 (1.0) |  |  |  |
| 37 | DRB3*01:01:02-*13:03:01-*05:05:01-*03:01:01;DRBX*null-*01:01:01-*01:01:01-*05:01:01 | 1 (0.2) | 1 (1.0) |  |  |  |
| 38 | DRB3*02:02:01-*03:01:01-*05:01:01-*02:01:01;DRB3*02:02:01-*11:01:01-*05:05:01-*03:01:01 | 0 | 1 (1.0) |  |  |  |
| 39 | DRB3*02:02:01-*03:01:01-*05:01:01-*02:01:01;DRB3*02:02:01-*11:01:01-*05:09-*03:01:01 | 0 | 1 (1.0) |  |  |  |
| 40 | DRB3*02:02:01-*03:01:01-*05:01:01-*02:01:01;DRB3*03:01:01-*13:02:01-*01:02:01-*06:04:01 | 2 (0.3) | 1 (1.0) |  |  |  |
| 41 | DRB3*02:02:01-*03:01:01-*05:01:01-*02:01:01;DRB4*01:03:01-*04:01:01-*03:02//03:03:01-*03:01:01 | 0 | 2 (2.0) |  | 3.57 | 3.55E-04 |
| 42 | DRB3*02:02:01-*03:01:01-*05:01:01-*02:01:01;DRB4*01:03:01-*04:02:01-*03:01:01-*03:02:01 | 0 | 1 (1.0) |  |  |  |
| 43 | DRB3*02:02:01-*03:01:01-*05:01:01-*02:01:01;DRB4*01:03:01-*04:05:01-*03:02//03:03:01-*03:02:01 | 0 | 1 (1.0) |  |  |  |
| 44 | DRB3*02:02:01-*03:01:01-*05:01:01-*02:01:01;DRB4*01:03:03-*04:04:01-*03:01:01-*03:02:01 | 0 | 1 (1.0) |  |  |  |
| 45 | DRB3*02:02:01-*11:01:01-*05:05:01-*03:01:01;DRB4*01:01:01//DRB4*01:01:01:01-*07:01:01-*02:01//02:01:01-*02:02:01 | 0 | 1 (1.0) |  |  |  |
| 46 | DRB3*02:02:01-*11:01:01-*05:05:01-*03:01:01;DRB5*02:02//DRB5*02:02:01-*16:01:01-*01:02:02-*05:02:01 | 2 (0.3) | 1 (1.0) |  |  |  |
| 47 | DRB3*02:02:01-*11:01:01-*05:05:01-*03:01:01;DRBX*null-*08:01:01-*04:01:01-*04:02:01 | 0 | 1 (1.0) |  |  |  |
| 48 | DRB3*02:02:01-*11:04:01-*05:05:01-*03:01:01;DRBX*null-*01:01:01-*01:01:01-*05:01:01 | 0 | 1 (1.0) |  |  |  |
| 49 | DRB3*02:02:01-*11:04:01-*05:05:01-*03:01:01;DRBX*null-*01:02:01-*01:01:02-*05:01:01 | 0 | 1 (1.0) |  |  |  |
| 50 | DRB3*02:02:01-*12:01:01-*05:05:01-*03:01:01;DRBX*null-*01:01:01-*01:01:01-*05:01:01 | 2 (0.2) | 1 (1.0) |  |  |  |
| 51 | DRB3*02:02:01-*13:05:01-*05:05:01-*03:01:01;DRB5*02:02//DRB5*02:02:01-*16:01:01-*01:02:02-*05:02:01 | 0 | 1 (1.0) |  |  |  |
| 52 | DRB3*02:02:01-*13:05:01-*05:05:01-*03:01:01;DRBX*null-*01:01:01-*01:01:01-*05:01:01 | 0 | 1 (1.0) |  |  |  |
| 53 | DRB3*02:02:01-*14:04//*14:04:01-*01:04:02-*05:03:01;DRB4*01:03:01-*04:02:01-*03:01:01-*03:02:01 | 0 | 1 (1.0) |  |  |  |
| 54 | DRB3*02:02:01-*14:54:01-*01:04:01-*05:03:01;DRB4*01:03:01-*04:06:02-*03:02//03:03:01-*04:02:01 | 0 | 1 (1.0) |  |  |  |
| 55 | DRB3*02:02:01-*14:54:01-*01:04:01-*05:03:01;DRB5*02:02//DRB5*02:02:01-*16:01:01-*01:02:02-*05:02:01 | 0 | 1 (1.0) |  |  |  |
| 56 | DRB3*03:01:01-*13:02:01-*01:02:01-*06:04:01;DRB4*01:03:01-*04:01:01-*03:01:01-*03:02:01 | 0 | 1 (1.0) |  |  |  |
| 57 | DRB3*03:01:01-*13:02:01-*01:02:01-*06:04:01:DRBX*null-*01:02:01-*01:01:02-*05:01:01 | 0 | 1 (1.0) |  |  |  |
| 58 | DRB3*03:01:01-*13:02:01-*01:02:01-*06:04:01;DRBX*null-*08:01:01-*04:01:01-*04:02:01 | 0 | 2 (2.0) |  | 3.57 | 3.55E-04 |
| 59 | DRB4*01:01:01//DRB4*01:01:01:01-*07:01:01-*02:01//02:01:01-*02:02:01;DRB5*01:02-*15:02:01-*01:03:01-*06:01:01 | 0 | 1 (1.0) |  |  |  |
| 60 | DRB4*01:01:01//DRB4*01:01:01:01-*07:01:01-*02:01//02:01:01-*03:03:02;DRB4*01:01:01//DRB4*01:01:01:01-*07:01:01-*02:01//02:01:01-*02:02:01 | 0 | 1 (1.0) |  |  |  |
| 61 | DRB4*01:03:01-*04:01:01-*03:02//03:02:01//03:03:01-*03:01:01;DRB4*01:03:01-*04:04:01-*03:01:01-*03:02:01 | 4 (0.5) | 1 (1.0) |  |  |  |
| 62 | DRB4*01:03:01-*04:02:01-*03:01:01-*03:02:01;DRBX*null-*01:02:01-*01:01:02-*05:01:01 | 0 | 1 (1.0) |  |  |  |
| 63 | DRB4*01:03:01-*04:03:01-*03:01:01-*03:02:01;DRB4*01:03:01-*04:02:01-*03:01:01-*03:02:01 | 0 | 1 (1.0) |  |  |  |
| 64 | DRB4*01:03:01-*04:03:01-*03:01:01-*03:02:01;DRBX*null-*08:01:01-*04:01:01-*04:02:01 | 0 | 1 (1.0) |  |  |  |
| 65 | DRB4*01:03:01-*04:04:01-*03:01:01-*03:02:01;DRB4*01:03:01-*04:01:01-*03:01:01-*03:02:01 | 0 | 1 (1.0) |  |  |  |
| 66 | DRB4*01:03:01-*04:04:01-*03:01:01-*03:02:01;DRB4*01:03:01-*04:11:01-*03:01:01-*03:02:01 | 0 | 1 (1.0) |  |  |  |
| 67 | DRB4*01:03:01-*04:04:01-*03:01:01-*03:02:01;DRBX*null-*01:01:01-*01:01:01-*05:01:01 | 0 | 1 (1.0) |  |  |  |
| 68 | DRB4*01:03:01-*07:01:01-*02:01//02:01:01-*02:02:01;DRBX*null-*01:01:01-*01:01:01-*05:01:01 | 2 (0.2) | 1 (1.0) |  |  |  |
| 69 | DRB5*02:02//DRB5*02:02:01-*16:01:01-*01:02:02-*05:02:01;DRB5*02:02//DRB5*02:02:01-*16:01:01-*01:02:02-*05:02:01 | 0 | 1 (1.0) |  |  |  |
| 70 | DRB5*02:02//DRB5*02:02:01-*16:01:01-*01:02:02-*05:02:01;DRBX*null-*01:02:01-*01:01:02-*05:01:01 | 0 | 1 (1.0) |  |  |  |
| 71 | DRBX*null-*01:01:01-*01:01:01-*05:01:01;DRBX*null-*01:01:01-*01:01:01-*05:01:01 | 9 (1.4) | 1 (1.0) | 1.00 | 0.33 | 7.39E-01 |

| Supplementary Table 2. Estimated haplotypic frequencies of HLA-DR -DQ genes among patients and controls, estimated odds ratios (OR) of individual alleles, estimated H-score (HS) measuring associations, and associated P-values. Haplotype variants marked in light grey (higher) and dark grey (lower) frequency differed between MS patients and controls. | | | | | | |
| --- | --- | --- | --- | --- | --- | --- |
| Hap | HLA-*DRB3, DRB4, DRB5-DRB1-DQA1-DQB1* | Control n (%) | MS n (%) | OR | HS | P-value |
| Base | DRB3*03:01:01-*13:02:01-*01:02:01-*06:04:01 | 51 (4.0) | 7 (3.5) | 1.00 | –0.35 | 7.25E-01 |
| 1 | DRB3*01:01:02-*03:01:01-*05:01:01-*02:01:01 | 125 (9.6) | 14 (7.0) | 1.13 | –1.20 | 2.29E-01 |
| 2 | DRB3*01-*01:01:01-*01:01:01-*05:01:01 | 1 (0.1) | 0 |  |  |  |
| 3 | DRB3*01-*13:01:01-*01:03:01-*06:03:01 | 1 (0.1) | 0 |  |  |  |
| 4 | DRB3*01:01:02-*03:01:01-*05:01:01-*02:01:08 | 8 (0.7) | 0 |  |  |  |
| 5 | DRB3*01:01:02-*11:01:01-*05:05:01-*03:01:01 | 2 (0.2) | 0 |  |  |  |
| 6 | DRB3*01:01:02-*13:01:01-*01:03:01-*06:03:01 | 54 (4.3) | 3 (1.5) | 0.45 | –1.97 | 4.88E-02, n.s |
| 7 | DRB3*01:01:02-*13:01:01-*01:10-*06:03:01 | 1 (0.1) | 0 |  |  |  |
| 8 | DRB3*01:01:02-*13:03:01-*05:05:01-*03:01:01 | 9 (0.7) | 1 (0.5) |  |  |  |
| 9 | DRB3*01:01:02-*13:03:01-*05:05:01-*03:09 | 1 (0.1) | 0 |  |  |  |
| 10 | DRB3*01:01:02-*14:02-*05:03-*03:01:01 | 3 (0.2) | 0 |  |  |  |
| 11 | DRB3*01:01:02-*14:12:01-*05:03-*03:01:01 | 1 (0.1) | 0 |  |  |  |
| 12 | DRB3*02:02:01-*03:01:01-*05:01:01-*02:01:01 | 19 (1.7) | 9 (4.5) | 4.43 | 2.62 | 8.84E-03 |
| 13 | DRB3*02:02:01-*03:01:01--*05:01:01-*02:01:08 | 1 (–) | 0 |  |  |  |
| 14 | DRB3*02:02:01-*08:01:01-*04:01:01-*04:02:01 | 1 (0.1) | 0 |  |  |  |
| 15 | DRB3*02:02:01-*08:04:01-*05:05:01-*03:01:01 | 1 (0.1) | 0 |  |  |  |
| 16 | DRB3*02:02:01-*11:01:01-*05:03-*03:01:01 | 2 (0.2) | 0 |  |  |  |
| 17 | DRB3*02:02:01-*11:01:01-*05:05:01-*03:01:01 | 57 (4.4) | 6 (3.0) | 0.82 | –0.92 | 3.59E-01 |
| 18 | DRB3*02:02:01-*11:01:01-*05:09-*03:01:01 | 1 (0.1) | 1 (0.5) |  |  |  |
| 19 | DRB3*02:02:01-*11:01:01-*05:10-*03:01:01 | 1 (0.1) | 0 |  |  |  |
| 20 | DRB3*02:02:01-*11:01:02-*01:02:01-*06:02:01 | 1 (0.1) | 0 |  |  |  |
| 21 | DRB3*02:02:01-*11:01:02-*05:05:01-*03:19:01//*03:19:01 | 1 (0.1) | 0 |  |  |  |
| 22 | DRB3*02:02:01-*11:02:01-*05:05:01-*03:01:01 | 3 (0.2) | 0 |  |  |  |
| 23 | DRB3*02:02:01-*11:02:01-*05:05:01-*03:19:01//*03:19:01 | 1 (0.1) | 0 |  |  |  |
| 24 | DRB3*02:02:01-*11:03//11:03:01-*05:05:01-*03:01:01 | 6 (0.5) | 0 |  |  |  |
| 25 | DRB3*02:02:01-*11:04:01-*01:03:01-*06:03:01 | 1 (0.1) | 0 |  |  |  |
| 26 | DRB3*02:02:01-*11:04:01-*05:03-*03:01:01 | 1 (–) | 0 |  |  |  |
| 27 | DRB3*02:02:01-*11:04:01-*05:05:01-*03:01:01 | 16 (1.3) | 5 (2.5) | 2.14 | 1.27 | 2.04E-01 |
| 28 | DRB3*02:02:01-*11:84:01-*05:05:01-*03:01:01 | 1 (0.1) | 0 |  |  |  |
| 29 | DRB3*02:02:01-*12:01:01-*05:05:01-*03:01:01 | 27 (2.1) | 2 (1.0) | 0.80 | –1.04 | 3.00E-01 |
| 30 | DRB3*02:02:01-*12:01:01-*05:05:01-*03:02:01 | 1 (0.1) | 0 |  |  |  |
| 31 | DRB3*02:02:01-*13:01:01-*01:03:01-*06:03:01 | 36 (2.7) | 2 (1.0) | 0.41 | –1.47 | 1.41E-01 |
| 32 | DRB3*02:02:01-*13:05:01-*05:05:01-*03:01:01 | 0 | 2 (1.0) |  |  |  |
| 33 | DRB3*02:02:01-*13:05:01-*05:05:01-*03:02:01 | 1 (0.1) | 0 |  |  |  |
| 34 | DRB3*02:02:01-*14:01:01-*01:01:01-*05:03:01 | 1 (0.1) | 0 |  |  |  |
| 35 | DRB3*02:02:01-*14:04//*14:04:01- *01:04:02-*05:03:01 | 0 | 1 (0.5) |  |  |  |
| 36 | DRB3*02:02:01-*14:04//*14:04:01--*01:01:01-*05:03:01 | 1 (–) | 0 |  |  |  |
| 37 | DRB3*02:02:01-*14:54:01-*01:01:01-*05:03:01 | 12 (0.9) | 0 |  |  |  |
| 38 | DRB3*02:02:01-*14:54:01-*01:04:01-*05:03:01 | 7 (0.6) | 2 (1.0) |  |  |  |
| 39 | DRB3*02:02:01-*14:54:01-*01:07-*05:03:01 | 1 (0.1) | 0 |  |  |  |
| 40 | DRB3*02:02:01-*14:54:01-*01:07Q-*05:03:01 | 1 (0.1) | 0 |  |  |  |
| 41 | DRB3*02:24-*14:01:01-*01:01:01-*05:03:01 | 3 (0.2) | 0 |  |  |  |
| 42 | DRB3*02:24-*14:01:01-*01:04:01-*05:03:01 | 1 (0.1) | 0 |  |  |  |
| 43 | DRB3*02:38-*13:02:01-*01:02:01-*06:04:01 | 1 (0.1) | 0 |  |  |  |
| 44 | DRB3*03:01:01-*11:01:02-*01:02:01-*06:02:01 | 1 (0.1) | 0 |  |  |  |
| 45 | DRB3*03:01:01-*13:02:01-*01:02:01- *06:09//*06:09:01 | 2 (0.2) | 1 (0.5) |  |  |  |
| 46 | DRB3*03:01:01-*13:02:01-*01:02:01-*05:01:01 | 2 (0.2) | 0 |  |  |  |
| 47 | DRB3*03:01:03-*12:02:01-*01:02:02-*05:02:01 | 1 (0.1) | 0 |  |  |  |
| 48 | DRB3*03:01:03-*12:02:01-*06:01:01-*03:01:01 | 2 (0.2) | 0 |  |  |  |
| 49 | DRB4*01:01:01//DRB4*01:01:01:01- *07:01:01-*02:01//02:01:01-*03:03:02 | 0 | 1 (0.5) |  |  |  |
| 50 | DRB4*01:01:01//DRB4*01:01:01:01-*04:01:01-*03:02//*03:03:01-*03:01:01 | 1 (0.1) | 0 |  |  |  |
| 51 | DRB4*01:01:01//DRB4*01:01:01:01-*07:01:01-*02:01//*02:01:01-*02:02:01 | 37 (2.8) | 9 (4.5) | 2.13 | 1.17 | 2.41E-01 |
| 52 | DRB4*01:02-*04:01:01-*03:02//03:03:01-*03:01:01 | 2 (0.2) | 0 |  |  |  |
| 53 | DRB4*01:03:01-*04:01:01- *03:02//03:03:01-*03:01:01 | 39 (3.0) | 6 (3.0) | 1.27 | 0.03 | 9.74E-01 |
| 54 | DRB4*01:03:01-*04:01:01-*03:01:01-*03:01:01 | 6 (0.5) | 0 |  |  |  |
| 55 | DRB4*01:03:01-*04:01:01-*03:01:01-*03:02:01 | 70 (5.6) | 5 (2.5) | 0.49 | -1.88 | 5.98E-02 |
| 56 | DRB4*01:03:01-*04:01:01-*03:02//*03:03:01-*03:01:03 | 1 (0.1) | 0 |  |  |  |
| 57 | DRB4*01:03:01-*04:01:01-*03:02//*03:03:01-*03:02:01 | 6 (0.5) | 0 |  |  |  |
| 58 | DRB4*01:03:01-*04:01:01-*05:05:01-*03:01:01 | 1 (0.1) | 0 |  |  |  |
| 59 | DRB4*01:03:01-*04:02:01-*03:01:01-*03:02:01 | 6 (0.5) | 6 (3.0) | 10.58 | 3.71 | 2.06E-04 |
| 60 | DRB4*01:03:01-*04:03:01-*03:01:01-*03:02:01 | 8 (0.5) | 5 (2.5) | 5.53 | 2.64 | 8.26E-03 |
| 61 | DRB4*01:03:01-*04:03:01-*03:01:01-*03:04 | 1 (0.1) | 0 |  |  |  |
| 62 | DRB4*01:03:01-*04:03:01-*03:01:01-*03:05:01 | 2 (0.2) | 0 |  |  |  |
| 63 | DRB4*01:03:01-*04:04:01-*03:01:01-*03:02:01 | 59 (4.7) | 6 (3.0) | 1.00 | –1.10 | 2.72E-01 |
| 64 | DRB4*01:03:01-*04:05:01- *03:02//*03:03:01-*03:02:01 | 3 (0.2) | 1 (0.5) |  |  |  |
| 65 | DRB4*01:03:01-*04:05:04--*03:01:01-*04:02:01 | 1 (–) | 0 |  |  |  |
| 66 | DRB4*01:03:01-*04:06:01-*03:02//*03:03:01-*04:02:01 | 1 (0.1) | 0 |  |  |  |
| 67 | DRB4*01:03:01-*04:06:02- *03:02//*03:03:01-*04:02:01 | 0 | 1 (0.5) |  |  |  |
| 68 | DRB4*01:03:01-*04:07:01-*03:01:01-*03:01:01 | 1 (0.1) | 0 |  |  |  |
| 69 | DRB4*01:03:01-*04:07:01-*03:01:01-*03:02:01 | 1 (0.1) | 0 |  |  |  |
| 70 | DRB4*01:03:01-*04:07:01-*03:02//*03:03:01-*03:01:01 | 9 (0.7) | 0 |  |  |  |
| 71 | DRB4*01:03:01-*04:08:01--*03:01:01-*03:02:01 | 1 (–) | 0 |  |  |  |
| 72 | DRB4*01:03:01-*04:08:01-*03:02//*03:03:01-*03:01:01 | 4 (0.4) | 0 |  |  |  |
| 73 | DRB4*01:03:01-*04:08:01-*03:02//*03:03:01-*03:04//*03:04:01 | 3 (0.2) | 0 |  |  |  |
| 74 | DRB4*01:03:01-*04:10:01-*03:02//*03:03:01-*04:02:01 | 1 (0.1) | 0 |  |  |  |
| 75 | DRB4*01:03:01-*04:11:01-*03:01:01*03:02:01 | 0 | 1 (0.5) |  |  |  |
| 76 | DRB4*01:03:01-*04:13-*03:02//*03:03:01-*03:01:01 | 1 (0.1) | 0 |  |  |  |
| 77 | DRB4*01:03:01-*07:01:01- *02:01//*02:01:01-*02:02:01 | 41 (3.2) | 1 (0.5) | 0.26 | –2.04 | 4.10E-02, n.s |
| 78 | DRB4*01:03:01-*07:01:01- *02:01//*02:01:01-*03:03:02 | 31 (2.5) | 0 |  | –2.25 | 2.41E-02 |
| 79 | DRB4*01:03:01-*09:01:02-*03:01:01-*03:03:02 | 2 (0.2) | 0 |  |  |  |
| 80 | DRB4*01:03:01-*09:01:02-*03:02//*03:03:01-*03:03:02 | 8 (0.6) | 0 |  |  |  |
| 81 | DRB4*01:03:01:02N-*04:02:01-*03:01:01-*03:02:01 | 1 (0.1) | 0 |  |  |  |
| 82 | DRB4*01:03:01:02N-*07:01:01-*02:01//*02:01:01-*03:03:02 | 5 (0.3) | 0 |  |  |  |
| 83 | DRB4*01:03:02-*04:04:01-*03:01:01-*03:02:01 | 1 (0.1) | 1 (0.5) |  |  |  |
| 84 | DRB4*01:03:02-*04:07:01-*03:01:01-*03:02:01 | 1 (0.1) | 0 |  |  |  |
| 85 | DRB4*01:03:02-*09:01:02-*03:02//03:03:01-*03:03:02 | 2 (0.2) | 0 |  |  |  |
| 86 | DRB4*01:03:03-*04:04:01-*03:01:01-*03:02:01 | 0 | 1 (0.5) |  |  |  |
| 87 | DRB5*01:01:01-*15:01:01-*01:02:01-*05:01:01 | 1 (0.1) | 0 |  |  |  |
| 88 | DRB5*01:01:01-*15:01:01-*01:02:01-*05:02:01 | 0 | 2 (1.0) |  |  |  |
| 89 | DRB5*01:01:01-*15:01:01-*01:02:01-*06:02:01 | 194 (15.3) | 55 (27.5) | 2.75 | 4.28 | 1.90E-05 |
| 90 | DRB5*01:01:01-*15:01:01-*01:02:01-*06:03:01 | 4 (0.3) | 2 (1.0) |  |  |  |
| 91 | DRB5*01:01:01-*15:01:01-*01:02:01-*06:14:01 | 1 (0.1) | 0 |  |  |  |
| 92 | DRB5*01:01:01-*15:01:01-*01:02:01-*06:187 | 0 | 1 (0.5) |  |  |  |
| 93 | DRB5*01:01:01-*15:01:01-*01:02:02-*05:02:01 | 1 (0.1) | 1 (0.5) |  |  |  |
| 94 | DRB5*01:01:01-*15:02:01-*01:01:01-*05:01:01 | 1 (0.1) | 0 |  |  |  |
| 95 | DRB5*01:01:01-*15:03:01-*01:02:01-*06:02:01 | 2 (0.2) | 1 (0.5) |  |  |  |
| 96 | DRB5*01:01:01-*15:06:01-*01:02:01-*05:02:01 | 0 | 1 (0.5) |  |  |  |
| 97 | DRB5*01:01:01-*16:09-*01:02:01-*06:02:01 | 1 (0.1) | 0 |  |  |  |
| 98 | DRB5*01:02-*15:02:01-*01:01:01-*05:01:24 | 1 (0.1) | 0 |  |  |  |
| 99 | DRB5*01:02-*15:02:01-*01:03:01-*06:01:01 | 6 (0.5) | 2 (1.0) |  |  |  |
| 100 | DRB5*01:08-*14:04//*14:04:01-*01:01:01-*05:03:01 | 1 (0.1) | 0 |  |  |  |
| 101 | DRB5*02:02//DRB5*02:02:01-*16:01:01- *01:02:02-*05:02:01 | 14 (1.1) | 8 (4.0) | 3.65 | 3.03 | 2.47E-03 |
| 102 | DRB5*02:02//DRB5*02:02:01-*16:01:01-*01:02:02-*06:02:01 | 1 (0.1) | 0 |  |  |  |
| 103 | DRB5*02:02//DRB5*02:02:01-*16:01:01-*01:02:02-*06:03:01 | 1 (0.1) | 0 |  |  |  |
| 104 | DRBX*null-*01:01:01-*01:01:01-*05:01:01 | 113 (8.9) | 9 (4.5) | 0.70 | –2.00 | 4.51E-02, n.s |
| 105 | DRBX*null-*01:01:01-*01:02:01-*05:04 | 3 (0.2) | 0 |  |  |  |
| 106 | DRBX*null-*01:02:01-*01:01:01-*05:01:01 | 9 (0.7) | 0 |  |  |  |
| 107 | DRBX*null-*01:02:01-*01:01:02-*05:01:01 | 4 (0.3) | 5 (2.5) | 12.46 | 3.70 | 2.18E-04 |
| 108 | DRBX*null-*01:03//*01:03:01-*01:01:01-*05:01:01 | 9 (0.7) | 0 |  |  |  |
| 109 | DRBX*null-*01:03//*01:03:01-*05:05:01-*03:01:01 | 2 (0.2) | 0 |  |  |  |
| 110 | DRBX*null-*07:01:01-*02:01//*02:01:01-*02:02:01 | 1 (0.1) | 0 |  |  |  |
| 111 | DRBX*null-*08:01:01-*04:01:01-*03:03:02 | 1 (0.1) | 0 |  |  |  |
| 112 | DRBX*null-*08:01:01-*04:01:01-*04:02:01 | 47 (3.7) | 10 (5.0) | 2.06 | 0.88 | 3.82E-01 |
| 113 | DRBX*null-*08:01:01-*04:02-*04:02:01 | 3 (0.2) | 1 (0.5) |  |  |  |
| 114 | DRBX*null-*08:01:01-*04:04-*04:02:01 | 1 (0.1) | 0 |  |  |  |
| 115 | DRBX*null-*08:02:01-*04:01:01-*04:02:01 | 3 (0.2) | 0 |  |  |  |
| 116 | DRBX*null-*08:03:02-*01:03:01-*06:01:01 | 3 (0.2) | 1 (0.5) |  |  |  |
| 117 | DRBX*null-*08:03:02-*06:01:01-*03:01:01 | 4 (0.3) | 0 |  |  |  |
| 118 | DRBX*null-*08:04:01-*05:05:01-*03:19:01//*03:19:01 | 1 (0.1) | 0 |  |  |  |
| 119 | DRBX*null-*10:01:01-*01:01:01-*05:01:01 | 7 (0.6) | 0 |  |  |  |
| 120 | DRBX*null-*10:01:01-*01:05:01-*05:01:01 | 1 (0.1) | 1 (0.5) |  |  |  |
| 121 | DRBX*null-*14:54:01-*01:01:01-*05:03:01 | 1 (0.1) | 0 |  |  |  |
| 122 | DRBX*null-*15:01:01-*01:02:01-*06:02:01 | 1 (0.1) | 0 |  |  |  |

| All | HLA-*DRB3*, *DRB4*, *DRB5* | Control n (%) | MS n (%) | OR | HS | P-value |  | Supplementary Table 3. Estimated allelic frequencies of HLA-*DRB3*, *DRB4*, *DRB5*, *-DRB1, -DQA1*, *-DQB1*, *-DPA1*, and *-DPB1* estimated odds ratios (OR) of individual alleles, estimated H-score (HS) measuring associations, and associated P-values. Allele variants marked in light grey (higher) and dark grey (lower) frequency differed between MS patients and controls. | | | | | | |
| --- | --- | --- | --- | --- | --- | --- | --- | --- | --- | --- | --- | --- | --- | --- |
|  |  |  |  |  |  |  |  |  |  |  |  |  |  |  |
| Base | DRB3*03:01:01 | 56 (4.4) | 8 (4.0) | 1.00 | –0.27 | 7.91E-01 |  |  |  |  |  |  |  |  |
| 1 | DRB3*01 | 2 (0.2) | 0 |  |  |  |  |  |  |  |  |  |  |  |
| 2 | DRB3*01:01:02 | 204 (16.0) | 18 (9.0) | 0.64 | –2.58 | 9.83E-03 |  |  |  |  |  |  |  |  |
| 3 | DRB3*02:02:01 | 202 (15.9) | 30 (15.0) | 1.08 | –0.32 | 7.49E-01 |  |  |  |  |  |  |  |  |
| 4 | DRB3*02:24 | 4 (0.3) | 0 |  |  |  |  |  |  |  |  |  |  |  |
| 5 | DRB3*02:38 | 1 (0.1) | 0 |  |  |  |  |  |  |  |  |  |  |  |
| 6 | DRB3*03:01:03 | 3 (0.2) | 0 |  |  |  |  |  |  |  |  |  |  |  |
| 7 | DRB4*01:01:01// DRB4*01:01:01:01 | 38 (3.0) | 10 (5.0) | 1.68 | 1.42 | 1.55E-01 |  |  |  |  |  |  |  |  |
| 8 | DRB4*01:02 | 2 (0.2) | 0 |  |  |  |  |  |  |  |  |  |  |  |
| 9 | DRB4*01:03:01 | 307 (24.1) | 32 (16.0) | 0.76 | –2.52 | 1.17E-02 |  |  |  |  |  |  |  |  |
| 10 | DRB4*01:03:01:02N | 6 (0.5) | 0 |  |  |  |  |  |  |  |  |  |  |  |
| 11 | DRB4*01:03:02 | 4 (0.3) | 1 (0.5) |  |  |  |  |  |  |  |  |  |  |  |
| 12 | DRB4*01:03:03 | 0 | 1 (0.5) |  |  |  |  |  |  |  |  |  |  |  |
| 13 | DRB5*01:01:01 | 205 (16.1) | 63 (31.5) | 2.30 | 5.25 | 1.52E-07 |  |  |  |  |  |  |  |  |
| 14 | DRB5*01:02 | 7 (0.6) | 2 (1.0) |  |  |  |  |  |  |  |  |  |  |  |
| 15 | DRB5*01:08 | 1 (0.1) | 0 |  |  |  |  |  |  |  |  |  |  |  |
| 16 | DRB5*02:02// DRB5*02:02:01 | 16 (1.3) | 8 (4.0) | 3.38 | 2.75 | 5.88E-03 |  |  |  |  |  |  |  |  |
| 17 | DRBX*null | 214 (16.8) | 27 (13.5) | 0.96 | –1.17 | 2.43E-01 |  |  |  |  |  |  |  |  |
|  |  |  |  |  |  |  |  |  |  |  |  |  |  |  |
| All | HLA-*DRB1* | Control n (%) | MS n (%) | OR | HS | P-value |  | All | *HLA-DQB1* | Control n (%) | MS n (%) | OR | HS | P-value |
| Base | *03:01:01 | 153 (12.0) | 23 (11.5) | 1.00 | 0.21 | 8.30E-01 |  | Base | *02:01:01 | 144 (11.3) | 23 (11.5) | 1.00 | 0.08 | 9.39E-01 |
| 1 | *01:01:01 | 117 (9.2) | 9 (4.5) | 0.51 | –2.12 | 3.36E-02, n.s |  | 1 | *02:01:08 | 9 (0.7) | 0 |  |  |  |
| 2 | *01:02:01 | 13 (1.0) | 5 (2.5) | 2.17 | 1.78 | 7.52E-02 |  | 2 | *02:02:01 | 78 (6.1) | 10 (5.0 | 0.74 | –0.62 | 5.37E-01 |
| 3 | *01:03//*01:03:01 | 11 (0.9) | 0 |  |  |  |  | 3 | *03:01:01 | 203 (16.0) | 23 (11.5) | 0.67 | –1.65 | 9.97E-02 |
| 4 | *04:01:01 | 126 (9.9) | 11 (5.5) | 0.50 | –2.00 | 4.56E-02, n.s |  | 4 | *03:01:03 | 1 (0.1) | 0 |  |  |  |
| 5 | *04:02:01 | 7 (0.6) | 6 (3.0) | 4.45 | 3.46 | 5.45E-04 |  | 5 | *03:02:01 | 160 (12.6) | 26 (13.0) | 0.92 | 0.17 | 8.65E-01 |
| 6 | *04:03:01 | 11 (0.9) | 5 (2.5) | 2.15 | 2.08 | 3.71E-02, n.s |  | 6 | *03:03:02 | 49 (3.9) | 1 (0.5) | 0.14 | –2.38 | 1.75E-02 |
| 7 | *04:04:01 | 61 (4.8) | 8 (4.0) | 0.84 | 0.50 | 6.17E-01 |  | 7 | *03:04//*03:04:01 | 4 (0.3) | 0 |  |  |  |
| 8 | *04:05:01 | 3 (0.2) | 1 (0.5) | 2.44 | 0.67 | 5.04E-01 |  | 8 | *03:05:01 | 2 (0.2) | 0 |  |  |  |
| 9 | *04:05:04 | 1 (0.1) | 0 |  |  |  |  | 9 | *03:09 | 1 (0.1) | 0 |  |  |  |
| 10 | *04:06:01 | 1 (0.1) | 0 |  |  |  |  | 10 | *03:19//*03:19:01 | 3 (0.2) | 0 |  |  |  |
| 11 | *04:06:02 | 0 | 1 (0.5) |  |  |  |  | 11 | *04:02:01 | 58 (4.6) | 12 (6.0) | 1.20 | 0.89 | 3.76E-01 |
| 12 | *04:07:01 | 12 (0.9) | 0 |  |  |  |  | 12 | *05:01:01 | 149 (11.7) | 15 (7.5) | 0.60 | –1.70 | 8.83E-02 |
| 13 | *04:08:01 | 8 (0.6) | 0 |  |  |  |  | 13 | *05:01:24 | 1 (0.1) | 0 |  |  |  |
| 14 | *04:10:01 | 1 (0.1) | 0 |  |  |  |  | 14 | *05:02:01 | 16 (1.3) | 12 (6.0) | 4.36 | 4.30 | 1.71E-05 |
| 15 | *04:11:01 | 0 | 1 (0.5) |  |  |  |  | 15 | *05:03:01 | 28 (2.2) | 3 (1.5) | 0.62 | –0.63 | 5.30E-01 |
| 16 | *04:13 | 1 (0.1) | 0 |  |  |  |  | 16 | *05:04 | 3 (0.2) | 0 |  |  |  |
| 17 | *07:01:01 | 114 (9.0) | 11 (5.5) | 0.60 | –1.61 | 1.08E-01 |  | 17 | *06:01:01 | 9 (0.7) | 3 (1.5) | 2.29 | 1.16 | 2.45E-01 |
| 18 | *08:01:01 | 53 (4.2) | 11 (5.5) | 1.32 | 0.85 | 3.95E-01 |  | 18 | *06:02:01 | 201 (15.8) | 56 (28.0) | 1.64 | 4.21 | 2.52E-05 |
| 19 | *08:02:01 | 3 (0.2) | 0 |  |  |  |  | 19 | *06:03:01 | 98 (7.7) | 7 (3.5) | 0.37 | –2.19 | 2.87E-02 |
| 20 | *08:03:02 | 7 (0.6) | 1 (0.5) |  |  |  |  | 20 | *06:04:01 | 52 (4.1) | 7 (3.5) | 0.70 | –0.40 | 6.87E-01 |
| 21 | *08:04:01 | 2 (0.2) | 0 |  |  |  |  | 21 | *06:09//*06:09:01 | 2 (0.2) | 1 (0.5) |  |  |  |
| 22 | *09:01:02 | 12 (0.9) | 0 |  |  |  |  | 22 | *06:14:01 | 1 (0.1) | 0 |  |  |  |
| 23 | *10:01:01 | 8 (0.6) | 1 (0.5) |  |  |  |  | 23 | *06:187 | 0 | 1 (0.5) |  |  |  |
| 24 | *11:01:01 | 63 (5.0) | 7 (3.5) | 0.67 | 0.91 | 3.65E-01 |  |  |  |  |  |  |  |  |
| 25 | *11:01:02 | 3 (0.2) | 0 |  |  |  |  | All | *HLA-DPA1* | Control n (%) | MS n (%) | OR | HS | P-value |
| 26 | *11:02:01 | 4 (0.3) | 0 |  |  |  |  | Base | *01:03:01 | 1072 (84.3) | 168 (84.0) | 1.00 | -0.10 | 9.23E-01 |
| 27 | *11:03//*11:03:01 | 6 (0.5) | 0 |  |  |  |  | 1 | *01:03:03 | 0 | 1 (0.5) |  |  |  |
| 28 | *11:04:01 | 18 (1.4) | 5 (2.5) | 1.56 | 1.11 | 2.67E-01 |  | 2 | *01:03:04 | 0 | 1 (0.5) |  |  |  |
| 29 | *11:84:01 | 1 (0.1) | 0 |  |  |  |  | 3 | *01:04 | 7 (0.6) | 2 (1.0) | 1.53 | 1.58 | 4.47E-01 |
| 30 | *12:01:01 | 28 (2.2) | 2 (1.0) | 0.44 | –1.09 | 2.75E-01 |  | 4 | *01:05 | 1 (0.1) | 0 |  |  |  |
| 31 | *12:02:01 | 3 (0.2) | 0 |  |  |  |  | 5 | *02:01:01 | 70 (5.5) | 19 (9.5) | 1.71 | 1.71 | 2.99E-02, n.s |
| 32 | *13:01:01 | 92 (7.2) | 5 (2.5) | 0.32 | –2.57 | 1.02E-02 |  | 6 | *02:01:02 | 64 (5.0) | 4 (2.0) | 0.39 | 0.39 | 7.10E-02 |
| 33 | *13:02:01 | 56 (4.4) | 8 (4.0) | 0.83 | 0.27 | 7.91E-01 |  | 7 | *02:01:04 | 3 (0.2) | 0 |  |  |  |
| 34 | *13:03:01 | 10 (0.8) | 1 (0.5) |  |  |  |  | 8 | *02:02:01 | 10 (0.8) | 0 |  |  |  |
| 35 | *13:05:01 | 1 (0.1) | 2 (1.0) |  |  |  |  | 9 | *02:02:02 | 34 (2.7) | 2 (1.0) | 0.35 | 0.35 | 1.61E-01 |
| 36 | *14:01:01 | 5 (0.4) | 0 |  |  |  |  | 10 | *02:06 | 6 (0.5) | 2 (1.0) |  |  |  |
| 37 | *14:02 | 3 (0.2) | 0 |  |  |  |  | 11 | *02:07:01 | 2 (0.2) | 0 |  |  |  |
| 38 | *14:04//*14:04:01 | 1 (0.1) | 1 (0.5) |  |  |  |  | 12 | *03:01//*03:01:01 | 2 (0.2) | 1 (0.5) |  |  |  |
| 39 | *14:12:01 | 1 (0.1) | 0 |  |  |  |  | 13 | *04:01 | 1 (0.1) | 0 |  |  |  |
| 40 | *14:54:01 | 22 (1.7) | 2 (1.0) | 0.50 | 0.73 | 4.64E-01 |  |  |  |  |  |  |  |  |
| 41 | *15:01:01 | 202 (15.9) | 61 (30.5) | 1.99 | 5.03 | 4.94E-07 |  | All | HLA-*DPB1* | Control n (%) | MS n (%) | OR | HS | P-value |
| 42 | *15:02:01 | 9 (0.7) | 2 (1.0) |  |  |  |  | Base | *02:01:02 | 178 (14.0) | 29 (14.5) | 1.00 | 0.19 | 8.47E-01 |
| 43 | *15:03:01 | 2 (0.2) | 1 (0.5) |  |  |  |  | 1 | *01:01:01 | 73 (5.7) | 4 (2.0) | 0.37 | –2.12 | 3.38E-02, n.s |
| 44 | *15:06:01 | 0 | 1 (0.5) |  |  |  |  | 2 | *01:01:02 | 1 (0.1) | 0 |  |  |  |
| 45 | *16:01:01 | 16 (1.3) | 8 (4.0) | 2.81 | 2.75 | 5.88E-03 |  | 3 | *02:02//*02:02:01 | 7 (0.6) | 2 (1.0) |  |  |  |
| 46 | *16:09 | 1 (0.1) | 0 |  |  |  |  | 4 | *03:01:01 | 123 (9.7) | 26 (13) | 1.43 | 1.46 | 1.45E-01 |
|  |  |  |  |  |  |  |  | 5 | *04:01:01 | 574 (45.1) | 77 (38.5) | 0.85 | –1.78 | 7.54E-02 |
| All | HLA-*DQA1* | Control n (%) | MS n (%) | OR | HS | P-value |  | 6 | *04:02:01 | 152 (11.9) | 28 (14) | 1.18 | 0.82 | 4.11E-01 |
| Base | *03:01:01 | 162 (12.7) | 25 (12.5) | 1.00 | –0.10 | 9.24E-01 |  | 7 | *04:02:03 | 0 | 1 (0.5) |  |  |  |
| 1 | *01:01:01 | 160 (12.6) | 9 (4.5) | 0.43 | –3.20 | 1.38E-03 |  | 8 | *05:01:01 | 27 (2.1) | 3 (1.5) | 0.63 | –0.57 | 5.71E-01 |
| 2 | *01:01:02 | 4 (0.3) | 5 (2.5) | 9.21 | 3.70 | 2.18E-04 |  | 9 | *06:01//*06:01:01 | 21 (1.7) | 2 (1.0) | 0.51 | –0.70 | 4.87E-01 |
| 3 | *01:02:01 | 265 (20.8) | 70 (35.0) | 1.85 | 4.46 | 8.15E-06 |  | 10 | *09:01:01 | 7 (0.6) | 4 (2.0) | 4.14 | 2.22 | 2.63E-02 |
| 4 | *01:02:02 | 18 (1.4) | 9 (4.5) | 3.15 | 2.94 | 3.28E-03 |  | 11 | *10:01//*10:01:01 | 10 (0.8) | 5 (2.5) | 3.41 | 2.26 | 2.41E-02, n.s |
| 5 | *01:03:01 | 101 (7.9) | 8 (4.0) | 0.53 | –2.02 | 4.35E-02 |  | 12 | *103:01v | 1 (0.1) | 0 |  |  |  |
| 6 | *01:04:01 | 8 (0.6) | 2 (1.0) |  |  |  |  | 13 | *104:01:01 | 4 (0.3) | 2 (1.0) |  |  |  |
| 7 | *01:04:02 | 0 | 1 (0.5) |  |  |  |  | 14 | *105:01:01 | 0 | 2 (1.0) |  |  |  |
| 8 | *01:05:01 | 1 (0.1) | 1 (0.5) |  |  |  |  | 15 | *11:01:01 | 11 (0.9) | 2 (1.0) |  |  |  |
| 9 | *01:07 | 1 (0.1) | 0 |  |  |  |  | 16 | *126:01:01 | 0 | 1 (0.5) |  |  |  |
| 10 | *01:07Q | 1 (0.1) | 0 |  |  |  |  | 17 | *13:01:01 | 15 (1.2) | 4 (2.0) | 1.61 | 0.96 | 3.36E-01 |
| 11 | *01:10 | 1 (0.1) | 0 |  |  |  |  | 18 | *14:01//*14:01:01 | 15 (1.2) | 2 (1.0) | 1.06 | –0.22 | 8.24E-01 |
| 12 | *02:01//*02:01:01 | 114 (9.0) | 11 (5.5) | 0.65 | –1.61 | 1.08E-01 |  | 19 | *15:01//*15:01:01 | 11 (0.9) | 2 (1.0) |  |  |  |
| 13 | *03:02//*03:03:01 | 81 (6.4) | 8 (4.0) | 0.72 | –1.29 | 1.98E-01 |  | 20 | *16:01//*16:01:01 | 8 (0.6) | 0 |  |  |  |
| 14 | *04:01:01 | 52 (4.1) | 10 (5.0) | 1.31 | 0.59 | 5.55E-01 |  | 21 | *17:01//*17:01:01 | 10 (0.8) | 1 (0.5) |  |  |  |
| 15 | *04:02 | 3 (0.2) | 1 (0.5) |  |  |  |  | 22 | *19:01:01 | 11 (0.9) | 0 |  |  |  |
| 16 | *04:04 | 1 (0.1) | 0 |  |  |  |  | 23 | *20:01:01 | 5 (0.4) | 1 (0.5) |  |  |  |
| 17 | *05:01:01 | 153 (12.0) | 23 (11.5) | 1.12 | –0.21 | 8.30E-01 |  | 24 | *21:01 | 1 (0.1) | 0 |  |  |  |
| 18 | *05:03 | 7 (0.6) | 0 |  |  |  |  | 25 | *23:01:01 | 4 (0.3) | 1 (0.5) |  |  |  |
| 19 | *05:05:01 | 131 (10.3) | 16 (8.0) | 0.81 | –1.01 | 3.12E-01 |  | 26 | *296:01 | 1 (0.1) | 0 |  |  |  |
| 20 | *05:09 | 1 (0.1) | 1 (0.5) |  |  |  |  | 27 | *40:01:01 | 1 (0.1) | 0 |  |  |  |
| 21 | *05:10 | 1 (0.1) | 0 |  |  |  |  | 28 | *45:01:00 | 0 | 1 (0.5) |  |  |  |
| 22 | *06:01:01 | 6 (0.5) | 0 |  |  |  |  | 29 | *678:01 | 1 (0.1) | 0 |  |  |  |
|  |  |  |  |  |  |  |  |  |  |  |  |  |  |  |
|  |  |  |  |  |  |  |  |  |  |  |  |  |  |  |
